# Trust and distrust in low‐income Michigan residents during the early COVID‐19 pandemic: A qualitative study

**DOI:** 10.1111/hex.13826

**Published:** 2023-07-14

**Authors:** Gavisha Waidyaratne, Eric Steinbrook, Shalini Roy, Annabella Opoku, Kaitlyn Jaffe, Susan Dorr Goold

**Affiliations:** ^1^ Department of Internal Medicine Ohio State University Wexner Medical Center Columbus Ohio USA; ^2^ University of Michigan Medical School Ann Arbor Michigan USA; ^3^ University of Cincinnati College of Medicine Cincinnati Ohio USA; ^4^ Department of Community Medicine and Population Health, College of Community Health Sciences University of Alabama Tuscaloosa Alabama USA; ^5^ Center for Bioethics and Social Sciences in Medicine University of Michigan Medical School Ann Arbor Michigan USA; ^6^ Center for Bioethics and Social Sciences in Medicine and Department of Internal Medicine University of Michigan Medical School Ann Arbor Michigan USA; ^7^ Department of Health Management and Policy, School of Public Health University of Michigan Ann Arbor Michigan USA

**Keywords:** COVID‐19, disparities, low‐income, Michigan, pandemic, trust

## Abstract

**Background:**

Trust and distrust have shaped health behaviour during the COVID‐19 pandemic. Since the start of the pandemic, misinformation and polarization eroded trust across the United States. In states like Michigan, pandemic restrictions led to significant unrest. Michiganders also faced disproportionate morbidity and mortality from COVID‐19 during this period.

**Objective:**

The objective of this qualitative study was to understand the individual experiences of trust in low‐income Michiganders during the early COVID‐19 pandemic.

**Participants:**

Twenty‐four participants at or below 200% of the federal poverty line who resided in Michigan were recruited for this study.

**Approach:**

Interviews were conducted during the winter of 2020 using a formal interview guide that addressed sources of information, perceptions of risk and exposure, protective behaviours and impacts of the pandemic at home, work and in receiving healthcare.

**Results:**

Thematic analysis showed that themes of trust and distrust emerged in multiple facets of our participants' experiences, including in the context of information sources, the behaviours of others, health, financial security, employment and overall safety. Trust and distrust in low‐income communities often stemmed from significant financial and economic vulnerabilities and instability in access to healthcare that was exacerbated in the pandemic. Furthermore, participant trust was shaped by internal (e.g., relationships with others) and external (e.g., source of information, social inequity) factors that influenced their perceptions and experiences during the pandemic.

**Conclusion:**

Trust has played an important role in many aspects of the experiences of low‐income communities during the COVID‐19 pandemic. This is important for clinicians to consider as COVID‐19 becomes endemic, and trust continues to impact patients' approaches to vaccines, testing and treatment options.

**Patient or Public Contribution:**

This study was designed and conducted with the assistance and input of the members of the DECIDERS Steering Committee, a diverse statewide network of community members in Michigan. The DECIDERS team allows community members to have a voice in the design and conduct of health research, and collaborates with researchers to improve health across the state of Michigan.

## 1 INTRODUCTION

The COVID‐19 global pandemic has had profound impacts on individuals and communities across the world. Early in the pandemic, Michigan was considered one of the hardest hit states with over 58,000 confirmed cases and 5600 deaths as of 1 June 2020.^1^ This was compounded by the social and political unrest that accompanied the ‘Stay Home, Stay Safe’ executive order signed by Governor Whitmer in March 2020. Although the executive order was effective in increasing social distancing behaviours, it also led to protests and deep mistrust in many Michiganders who were influenced by political polarization and misinformation surrounding the pandemic.^2^, ^3^

There has been considerable research examining the impact of COVID‐19 on trust amongst various populations around the world. In general, studies have documented higher levels of trust in healthcare providers and federal public health institutions compared to news media and social media.^4^ However, exposure to widespread misinformation, ‘fake news’, and contradictory information has led to higher levels of mistrust.^5^, ^6^ COVID‐related protective behaviours are also impacted by individual politics and partisan beliefs.^7^ Trust in politicians and confidence in national health agencies appear to correlate with individual compliance with personal and social mitigation behaviours.^8^ Similarly, studies have shown that risk perception, trust and general worldview impact individual acceptance of measures implemented during the pandemic.^9^, ^10^

There has been a disproportionate burden of COVID‐19‐related mortality and morbidity amongst Black and Native American communities in the United States.^11^ African American and Hispanic/Latinx communities also have lower levels of trust around vaccines, childcare/education policies and general pandemic information.^12^, ^13^ Meanwhile, rural communities across the United States, which face barriers to access to healthcare and health insurance, have also been shown to have higher rates of COVID‐19 vaccine hesitancy compared to urban populations in the United States.^14^ Research on the experiences of low‐income communities have shown that trust varies greatly based on location.^15^, ^16^ In Michigan specifically, the ongoing Flint Water Crisis has further exacerbated mistrust of government efforts to protect and preserve health amongst African American Michiganders.^17^, ^18^ However, there is limited data on how COVID‐19 has impacted trust perceptions in low‐income communities in Michigan.

The objective of this study was to understand the views and experiences of low‐income Michiganders during the early COVID‐19 pandemic, including sources of information, perceptions of risk and exposure, protective behaviours, impacts at home/work/healthcare and the impact of the pandemic on existing health barriers. This study provides novel insights into the prominent role of trust in most of these domains.

## 2 METHODS

Participants were recruited in the fall of 2020. An open recruitment strategy used ads and flyers in English and Spanish distributed on online forums (Craigslist, institutional health research website, etc.), emails, texts and community partners. Interested individuals were contacted by investigators over the phone for further screening. Inclusion criteria required participants to be Michigan residents, with a household income at or below 200% of the 2020 Federal Poverty Guidelines. Individuals under the age of 18 and over the age of 65 were excluded. In total, 155 prospective participants were screened through a series of 12 questions. Participants were selected to achieve a balance across geographic regions, age, gender, race and ethnicity. Counties were identified as rural or urban based on the Michigan Department of Community Health's 2012 Rural and Urban County Classification.^19^ In total, 24 participants were recruited for the study and scheduled for interviews. Informed consent was obtained by each recruited participant before the interview date.

All participants underwent a semistructured phone interview during the winter of 2020. Interviews lasted approximately 40 min to 1 h and were conducted in English or Spanish, using an interview guide. Interviews used open‐ended questions to address a wide range of topics including risk perception, views on public health policies, sources of information, protective behaviours, barriers to care and impact on resources/finances. All participants were provided monetary compensation via Visa debit card for their participation in the study. The study was approved by the Institutional Review Board of the University of Michigan.

Interviews were audio‐recorded and transcribed verbatim. One interview had audio recording quality too poor to permit reliable transcription. Spanish interpreters assisted with conducting Spanish language interviews and translating Spanish interviews before transcription. Underlying themes were identified and used to develop an initial code structure which was reviewed and edited, primarily through the addition of themes and subthemes during analysis. Analysts tagged excerpts with themes and subthemes that were incorporated into a final codebook that included inclusion and exclusion criteria and examples. Five team members independently performed thematic analyses on the interview scripts using Dedoose version 8.3.45, with 2–3 analysts per interview. Divergent coding was compared between investigators until a consensus could be reached.

## 3 RESULTS

Of the 24 participants with transcribed interviews, 11 (45.8%) were male and 13 (54.2%) were female (Table 1). Two‐thirds of participants had residence in urban counties and a third resided in rural counties. A total of 18 different Michigan counties were represented in this study. Participants' ages ranged from 20 to 65 years. Participants reported their race/ethnicity as White (8, 33.3%), Black (8, 33.3%), Hispanic (6, 25%), Arab/Chaldean (1, 4.2%) or Native American (1, 4.2%). Four interviews were conducted in Spanish. More than half of participants had a household income of <$2000 per month.

**Table 1:** Participant demographics.

	Number (%)
Sex	
Male	11 (45.8)
Female	13 (54.2)
County of Residence	
Urban	16 (66.7)
Rural	8 (33.3)
Age (years)	
18–29	6 (25)
30–39	8 (33.3)
40–49	5 (20.8)
50–59	3 (12.5)
60–65	2 (8.3)
Race/ethnicity	
White	8 (33.3)
Black	8 (33.3)
Hispanic	6 (25)
Arab/Chaldean	1 (4.2)
Native American	1 (4.2)
Household income ($/month)	
<1000	6 (25.0)
1000–1999	8 (33.3)
2000–2999	7 (29.2)
3000–3999	2 (8.3)
≥4000	1 (4.2)

Within our interviews, subthemes of trust and distrust were identified in multiple aspects of the participants' pandemic experiences. Interviewees expressed concerns with regard to whom they were trusting, when to trust others, vulnerability and risk and influencers/justifiers of trust (Table 2).

**Table 2:** Interview discussion themes related to trust and supporting quotes.

Who is being trusted?—sources of information, friends, family, coworkers, employers, etc.	60 year White male, urban—Yeah, it's tough. [My daughter] actually is working…a frontline worker…She had, you know, safety and all that. But, you know, you just have to know who's she been talking to all day. You know, she went to hug me…I want to hug her because I love her, but at the same aspect…Who knows who she's been talking to, you know? () 26 year Black male, rural—I actually feel safe because my place of work is actually adhering to these COVID‐19 protocols…it doesn't mean that I'm…100% safe, you know, because things happen. We are human beings, and we are prone to error, too. There is nothing like 100% satisfaction, but…I feel safe in my place of work. 54 year White male, urban—My family doctor, I've been seeing her for years…She's one of the best people I ever met in my life…She would see me if I didn't have insurance, you know?… If she thought I was sick and I needed help, she would be there for me. 37 year Hispanic female, urban—I definitely feel like medical professionals, scientists…For example, like I know I do read about what like Dr. Fauci says and stuff like that…I find those folks to be trustworthy.
What are others being trusted with?—health, workplace safety, financial/job security, etc.	25 year Black female, urban—We don't like bring people into our home. You know, our father has…a health issue. So I've heard that people with health issues are more at risk of…not being able to fight the virus. So we decided not to bring people around. You don't know where they've been. You can't force them to protect themselves. So if I want to meet someone and someone wants to meet me, I just prefer to see them outside of our home. 40 year Black male, urban—I feel unsafe [at work] because when you're at the construction site, some people are not that cautious. They don't keep any social distancing…They drop their masks below their nose. So you just have to be cautious on your own for your own sake. 47 year Native American female, urban—INTERVIEWER: How do you feel about [your son going to work]? PARTICIPANT: I feel okay because the owner [of the company] is a family member of mine…So I felt pretty safe knowing that my son was working with him.
What causes the risk and vulnerability that force individuals to trust others?—financial/economic vulnerability, fear, health insurance, etc.	32 year Arab/Chaldean female, rural—Both my husband and I…were reduced in our working schedules. So it has been hard for us economically to provide for our kids, for bus fare and other bills and electricity bills. We have to look for other odd jobs to do it. 40 year Black male, urban—For me, I was laid off…They told us that we would be hired back definitely…We were told they would update us before the new year so that we can go back to work in January. I'm in lay‐off right now because everything is still down. [INTERVIEWER: And how long have you been in layoff?] 5 months. [What has that been like for you and your family?] Very tough. 30 year Black female, urban—You're not sure what will happen. You might die or you might survive. Second, you don't know…what should happen if indeed you recover from the thing. You hear of people who have recovered, but they actually have medical complications…You're not certain about anything, about the whole thing. It's very scary.
What influences and justifies trust?—consistency, reputation, popularity, transparency, verifiability, social unity, generational differences	37 year Hispanic female, urban—[MSNBC] doesn't seem like they're trying to pick a side. It seems like they're trying to deliver the news to you, but they're also journalists and news media. 56 year White female, rural—When all the agencies are reporting the same thing…It seems to be pretty trustworthy, but, you know, you do want to trust the news media, especially with something as serious as this. 27 year White female, urban—I'm the type of person that likes to gather a lot of sources and information and then kind of go about the census with it. So I don't really have anything like, ‘yeah, I love this news station. This is where I always look for news’. 65 year White male, rural—What has this world become? You know, 80 years, and I'm living like they were back in the depression. We have trains, we have planes, we have nuclear stuff. We're sending rockets to the moon…Mars and spending billions and trillions of dollars in outer space, and yet the poor people are still dying every day because we're not important enough.

### 3.1 Who to trust?

Many participants discussed whom or what they trusted (the trustee) during the early days of the pandemic. In many of our interviews, trust or distrust was directed towards sources of information. Interviewees used many different sources to acquire information during the pandemic, including news (online and TV), social media, radio, public health institutions and medical providers. Many participants expressed polarizing views towards certain sources of information. One 48‐year‐old rural female shared their perspective on the news:

I haven't watched the news literally in years because I think it's garbage, but when all this was creeping up…I started watching Democracy Now and the Daily Show with Trevor Noah, and they got me all scared and confused like everybody else, and then I realized they were absolutely lying and I haven't watched them since, and they need to go to jail

In contrast, participants consistently expressed more trust towards information provided by public health institutions and healthcare providers:

[Information from the] local department [is] a bit more clear…You're around to see what's going on. So, I guess I'll trust more the people that are close to me…than I would even trust the state department because with the local department…I'm around there to just see what's going on, and I'm able to verify if it's true or not.

In addition to sources of information, some participants described their trust or distrust in other individuals around them, including friends, family, coworkers and employers. Many of these views were in light of whether others were adhering to protective behaviours (mask wearing, social distancing, sanitizing, etc.). One interviewee, a 33‐year‐old White urban male, described:

Even when there's not a pandemic, [my family] generally don't come into contact with a whole lot of other people…And it's similar with friends. It's mostly the same group of friends who kind of all hang out…They would get tested if they thought they were exposed, and everybody's very responsible about it…I trust everyone that I have hung out with

### 3.2 Trust with what?

When discussing trust, participants described various trust objects, or what their trustees were being trusted with.^20^ Many participants described the experience of trusting others with either their own health or the health of loved ones. This theme emerged in the context of trusting others' (e.g., friends and family) adherence to COVID‐19‐related precautions so as not to spread the virus and in the context of accessing healthcare. A 41‐year‐old Spanish‐speaking Hispanic woman described the experience of trusting the care of her husband, sick with COVID‐19, to the hospital:

Because that's the first thing they show on the news. That so many people came [to the hospital] and they didn't come out… But I told [my husband] he needs to have faith. And I told him he needs to have faith that he will make it out and everything will be okay

Some interviewees shared concerns about trusting employers and coworkers with providing safe working conditions at their place of employment during the pandemic with regard to social distancing, access to masks/hand sanitizer, etc (Table 2). In addition, some participants described distrusting others with their financial or job security, particularly with regard to social support programs. One mother of three, a 34‐year‐old Black female remarked:

I just feel like Michigan can do a lot more letting people know the resources that they have available for people during the COVID…I just feel like there's a disconnect in letting people know the resources…Maybe they just don't want everybody trying to get all the benefits

### 3.3 Vulnerability and risk

Perceptions of vulnerability and risk emerged as central themes in every interview. Many interviewees described feeling fear during the early pandemic, including the fear of death, fear of medical complications, and fear of COVID‐19 exposure to self or family (Table 2). Several participants also expressed fear around a lack of health insurance and vulnerability related to losing insurance after a job loss.

Financial vulnerability was a common theme that presented in the forms of bankruptcy, filing for disability, furlough without pay, pay cuts, relying on others for financial support and difficulty paying rent/bills. Many interviewees described the experience of vulnerability in employment including job loss, difficulty finding employment, decreased work and feeling the need to work. For example, a 30‐year‐old urban female participant reported:

I used to work in a beauty shop…It closed. So I lost my job, but I'm getting support from my husband and relatives…it has drained me financially and has affected me mentally. [The beauty shop] opened with just a few [staff]. So for us, we are waiting, but we should go back

### 3.4 Influencers and justifiers of trust

When discussing the pandemic, participants also described what qualities influenced and justified their trust. Many interviewees elaborated on factors that affected their trust in sources of information. Qualities that seemed to increase the trustworthiness of sources included consistency, transparency, accuracy, verifiability and popularity (Table 2). For example, one 34‐year‐old urban female explained:

I would try to look at the videos that had millions and thousands of views and likes and things… I can't tell you offhand the exact ones because I'm just a very big Internet browser…but the ones that didn't have a lot of views, I didn't really think they were trustworthy

For some participants, their relationship with others around them justified their trust or distrust. A common theme that bred distrust was generational differences. Older participants described wariness towards younger generations due to perceived differences in responses to the pandemic. A 60‐year‐old White male from Clinton County (urban) said:

I see people out there struggling all the time…I'm always trying to help out…It shows you the old school people how they are, you know? [Younger people] don't think like the older people used to, you know? It's all about them. They just won't bother [to help others]

Other participants described how social inequity and disunity contributed to their overall feelings of distrust during the pandemic. One interviewee, a 65‐year‐old rural male, described the following perspective:

Everybody talks about rights…yeah, you have the right to go out there and not wear a mask if you don't want to. That's your right, but don't jeopardize the person standing next to you because you want to turn around and use your rights… what about the person next to you, you know? Be human… Have some humanity. Think about that person next to you. Think about your neighbors, your grandparents…your siblings

## 4 DISCUSSION

Numerous studies have described how trust has had a critical impact on the COVID‐19 pandemic. Trust has shaped individuals' COVID‐related behaviours, where greater trust in the government's pandemic response is associated with higher adoption of health behaviours.^9^, ^21^ However, misinformation, inconsistency and partisan politics have led to an increasingly fragile state of trust and distrust around COVID‐19 in the United States.^5^, ^6^ This is further compounded in racial and ethnic minority communities, which face health disparities, have fewer resources, and experience greater distrust towards the healthcare system and government due to histories of systemic racism and discrimination.^17^ The objective of our study and analysis was to examine how trust and distrust influenced the experiences of a diverse array of low‐income individuals in Michigan during the early COVID‐19 pandemic. Our study is the first of its kind in Michigan and demonstrates the breadth with which trust has influenced the lives of our participants.

While we expected trust and distrust to arise with regard to sources of information, these themes also emerged in many other pandemic experiences. Trust and distrust were entwined in discussions around the behaviours of others, health (both self and others), financial and job security and overall safety. Much of our interviewees' trust and distrust was specifically directed towards other individuals including friends, family, coworkers, employers and medical professionals.

Our participants' trust (or distrust) was often intense and stemmed from major vulnerabilities and instability. Participants often described structural vulnerabilities that were exacerbated during the COVID‐19 pandemic; these ranged widely from financial and economic (bankruptcy, pay cuts, difficulty paying rent/bills, job loss) to health‐related (lack of health insurance). These feelings of vulnerability forced participants into trusting family or friends for financial support during the early pandemic. Furthermore, trust was influenced by a variety of external factors including consistency, reputation, popularity, transparency, accuracy and verifiability. In contrast, distrust was sowed by factors such as unverifiable claims in the media, generational differences and social inequity and disunity.

While our study focused on the experiences of trust during the early months of the pandemic, other studies have shown that trust continues to have a considerable impact on the trajectory of the pandemic with regard to vaccines, testing, and treatment options. A large observational study by Bollyky et al. showed that from 2020 to 2022, American states with a greater proportion of people expressing interpersonal trust were associated with lower rates of COVID‐19 infection and death, as well as higher rates of vaccination.^22^ Other studies have found that trust in government, social trust, trust in physicians and mistrust of public health institutions and scientists are associated with vaccination intention and rates.^23^, ^24^ These findings are consistent with many of the expressions of trust and distrust made by our participants during the early pandemic.

There were several limitations to this study. Our cohort allowed us the opportunity to hear a range of experiences from individuals who were likely to face greater risks due to disadvantages; our constrained sample size may have kept us from hearing other important experiences and did not allow to generalize our findings to others with shared backgrounds. Data collection was limited to the early months of the pandemic; topics such as vaccines that became widely available after the end of the study were not discussed in depth during the interviews. However, while the role of trust in vaccine hesitancy has been widely investigated in studies like Bollyky et al., our early findings provide useful insight into how trust and distrust affected choices and behaviours regarding many other aspects of the pandemic (sources of information, social distancing, mask wearing, etc).

Despite the limitations of our study, the significance of trust and distrust during the early COVID‐19 pandemic in low‐income communities is evident. The uncertainty caused by the pandemic will continue as COVID‐19 becomes endemic, and distrust spills over into other areas of public health and healthcare (e.g., vaccine scepticism, misinformation); this is an important consideration for clinicians approaching patient care. While our study investigated trust during the early COVID‐19 pandemic, further research should examine how trust in low‐income communities has evolved, what interventions or factors increase or undermine trust, and how trust influences not only vaccination intentions, but also other health behaviours recommended by medical providers, scientists, and public health institutions.

## AUTHOR CONTRIBUTIONS

Gavisha Waidyaratne, Eric Steinbrook and Susan Dorr Goold contributed to the design and implementation of the research. Gavisha Waidyaratne, Eric Steinbrook, Shalini Roy, Annabella Opoku, Kaitlyn Jaffe and Susan Dorr Goold contributed to the analysis of the results and to the writing of the manuscript.

## CONFLICT OF INTEREST STATEMENT

The authors declare no conflict of interest.

## ETHICS STATEMENT

The study was approved by the Institutional Review Board of the University of Michigan.

## Data Availability

The data that support the findings of this study are available on request from the corresponding author. The data are not publicly available due to privacy or ethical restrictions.
